# Multiple Giant Coronary Artery Aneurysms Surgically Treated with Bypass Grafting: A Challenging Rarity

**DOI:** 10.1155/2018/2096902

**Published:** 2018-08-29

**Authors:** Kostas Kostopanagiotou, Aikaterini Poulou, Andrew Chatzis, Mazen Khoury

**Affiliations:** ^1^Thoracic Surgery Department, Attikon University Hospital, Athens, Greece; ^2^Cardiac Surgery Department, Onassis Cardiac Surgery Centre, Athens, Greece

## Abstract

Coronary artery aneurysms are encountered in daily cardiology practise but multiple giant-sized coronary artery aneurysms are extremely rare. We present an illustrative case of multiple giant aneurysms located throughout the coronary system (left main stem and all left, right, and circumflex branches) in a 57-year-old male with acute coronary syndrome. The case was managed successfully with on-pump quadruple coronary artery bypass grafting. To our knowledge, few cases of multiple giant aneurysms in all coronary vessels have been reported.

## 1. Introduction

In the current era, multiple diagnostic tools are available in everyday cardiology practise, and rare findings of the past are encountered and published more often. The presence of coronary artery aneurysms is a well-described entity as well as their occasional giant size. However, multiple giant size aneurysms involving all the coronary arteries are extremely rare and may present unique interesting clinical images and also present as a diagnostic and management challenge.

## 2. Case Presentation

**Figure 1:**
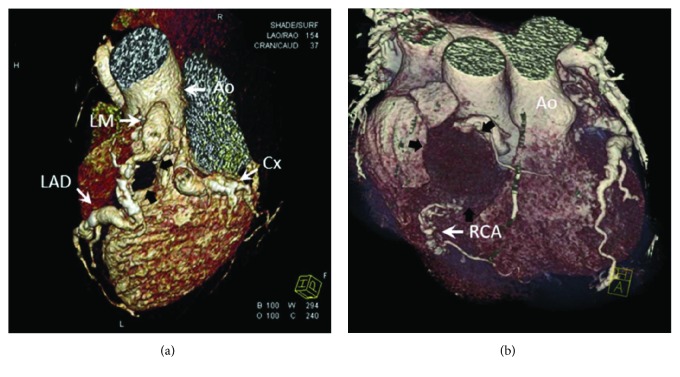
A 3D reconstruction image of the heart. In (a), the white arrows show the left coronary system with aneurysmal main stem and LAD (black arrows) and circumflex artery. In (b), the black arrows show the spherical giant aneurysm of the RCA which measures more than four centimeters.

A 57-year-old Caucasian male presented at the emergency department with acute chest pain and uncontrolled hypertension of 180/100 mmHg. Past medical history included ongoing smoking, high body mass index (BMI > 30), arterial hypertension (150/95 mmHg at rest), hyperlipidemia (serum LDL levels > 230 mg/dL), and a failed ablation for atrial fibrillation eight years previously. Medications at home were clopidogrel, acenocoumarol, oral amiodarone, a b-blocker, and a calcium-channel blocker, but it was unclear if these were taken as instructed. There was no history of vasculitis or other collagen diseases. The ECG on admission did not show ischemic abnormalities, and blood biochemistry led to the diagnosis of myocardial infarction with mildly elevated troponin level (0.4 ng/mL max value 0.1 ng/mL). His chest X-ray was unremarkable. Echocardiography was performed. The anterior-basic, anterior-septal, and the anterior-lateral portions of the myocardium of the left ventricle were hypokinetic. The left ventricular ejection fraction was calculated at 45%. No abnormal structural findings or mediastinal masses were noticeable. Coronary angiography was performed, and multiple large (over one centimeter) arterial aneurysms on both left and right coronary arteries were identified. In detail, the right coronary artery (RCA) was dilated just after its origin and along the vessel's entire length to a maximum diameter up to 43 mm and presenting with a thrombosed lumen, significant postaneurysm stenosis, and retrograde flow from the left coronary artery. The left main stem (LM) artery was 11.5 mm and gradually dilated in continuation with the anterior descending branch (LAD) to a maximum of 28 mm in diameter, but there was a patent lumen of internal diameter 9.3 mm distally. The circumflex artery (LCx) was dilated from 8.5 mm up to 12 mm for most of its tortuous length. A subsequent multislice computed tomography scan with intravenous contrast was performed to exclude the presence of other extracardiac aneurysms including the brain. The 3D reconstruction images obtained from the CT angiogram revealed the very interesting morphology of these GCAAs which was clinically overlooked in echocardiography at the acute assessment (Figure 1). Myocardial revascularization was indicated due to these findings, and the patient was referred for cardiac surgery in the same week.

**Figure 2:**
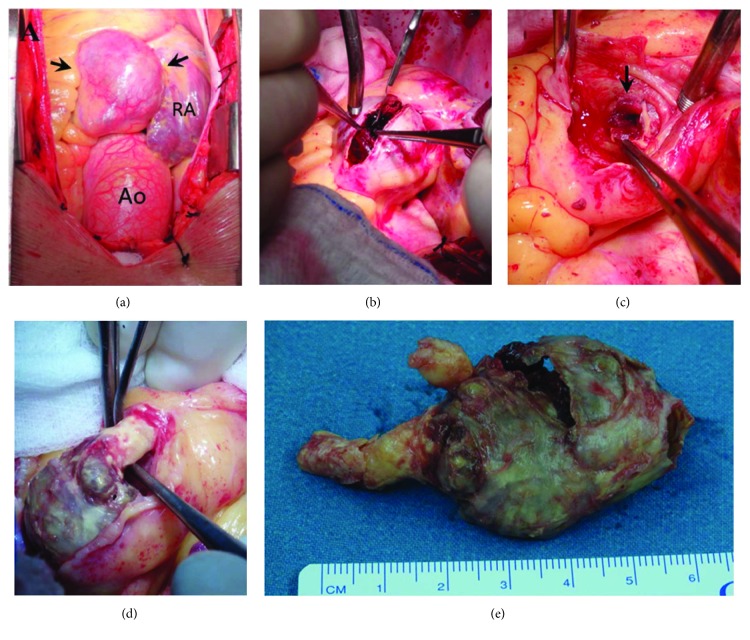
Intraoperative images. The black arrows in (a) show the giant CAA which is larger than the nearby aortic root (Ao) and the right atrium (RA). In (b), the RCA aneurysm is dissected open, and after thrombus debridement, the stenotic vessel lumen is indicated with a black arrow in (c). In (d), the main stem and LAD are dissected open and endarterectomy is performed. In (e), the atherosclerotic cast is measured before it was sent for histopathology and microbiology investigations.

Standard median sternotomy was performed. Upon opening the pericardium, the operating field was dominated by large-sized coronaries and especially the RCA one which appeared larger than the aortic root (Figure 2(a)). There were no actual pressure effects on the cardiac chambers or the valvular mechanism. These aneurysms resembled hard extracardiac masses. The decision to explore the aneurysms and bypass them was taken. Bilateral internal mammary arteries were harvested for use as grafts. Cardiopulmonary bypass (CPB) was established, and cold blood-based cardioplegia (4 : 1) was delivered in antegrade initially and retrograde fashion through the coronary sinus as per standard. All aneurysms were sequentially dissected open starting with the largest RCA (Figure 2(b)). Thrombus material and calcified plaques were removed (Figure 2(c)). Samples were taken for histopathology, serology, and microbiology examination. This was repeated at the aneurysms of the left coronary artery and the circumflex artery. The LAD was dissected open distally to the stem, and endarterectomy was performed to provide an area for distal anastomosis (Figure 2(d)). A large calcified cast was sent for investigation (Figure 2(e)). Interestingly, all the distal vessel lumens had postaneurysmal stenosis as shown on Figure 2(c). The dissected portions of the coronaries were ligated proximally and distally with prolene 5-0 sutures prior to the execution of the bypass. The pedicled left internal mammary artery (LIMA) was anastomosed to the LAD, the right internal mammary artery (RIMA) as a free graft to the marginal branch of the circumflex artery. Two saphenous vein grafts (previously harvested) were anastomosed to the RCA and the intermediate branch of the (LCx) artery, respectively. Aortic cross-clamp time was 224 min and total bypass time was 252 min. Intraoperative transfusion requirements were 2 units of red blood cells and 5 units of fresh-frozen plasma. There were no intraoperative complications, and he was transferred to the ICU on inotropic support with dobutamine 5 *μ*g/kg/min weaned after eight hours and extubated after twelve hours. The patient was discharged on the sixth postoperative day after an uneventful stay. He was prescribed a long-life medication with oral salicylic acid 100 mg daily and acenocoumarol to target INR 2.0-3.0. The histopathological results were negative for arteritis, autoimmune pathology, or infection, and only changes related to coronary artery disease, i.e., media disruption with focal calcification and hyalinization with lipid and cholesterol crystal deposits were found. Follow-up at one and six months and thereafter a year with echocardiography was satisfactory as the patient remains pain-free and in full physical activity.

## 3. Discussion

An increasing number of scientific papers describing coronary artery aneurysms have been published over the last years. The reasons for this trend include (a) advanced modern diagnostic modalities in cardiology, (b) newer higher quality imaging equipment, and (c) broader access to cardiology services for the general population in most places around the world. Thus, more data have emerged on the pathology, management, and outcome of CAAs. The peculiarity of our case report is the multiplicity and extent of the CAAs which involve the majority of the coronary arterial network.

The coronary arteries are defined as aneurysmatic when their diameter exceeds the diameter of the adjacent normal vessel by one and a half times according to Swaye et al. [1]. The size which characterizes giants CAAs is liberal, but increases in diameter above 20 mm have been given the name giant [2]. The general natural history of CAAs attributes most cases to atherosclerosis, and Demopoulos et al. refer to CAAs as a variant of obstructive coronary artery disease [3]. Age and male gender are contributing factors [1, 4]. A different patient group includes those with congenital dysplasia or systemic diseases (Kawasaki disease and Takayasu arteritis) [4, 5]. Those who underwent a percutaneous vascular intervention and iatrogenic vessel injury have been caused to have pseudoaneurysms and not actual CAAs. CAAs are a common finding mostly in patients undergoing coronary angiography (prevalence up to 5%), are located mostly on the right coronary artery, can be multiple in a fourth of the diagnoses, and their management depends on the size and clinical presentation. Fewer in number but quite descriptive publications exist regarding the giant CAAs which are extremely rare as Li et al. identified only 6 cases among 30,268 adult cardiac surgery patients in a single-centre retrospective study which reflects an incidence rate of 0.01% [6]. A focused study on giant CAAs has been published by Keyser et al. with 27 patients traced over the Pubmed and Medline databases whose diameter was over 5 cm. For most of the patients in the series, surgery was performed successfully [7]. A question is raised why the giant sizes are worth mentioning in the literature apart from the interesting illustrations. The fact is that they present an independent predictor of mortality with an overall 5-year survival of 71% due to potentially fatal complications of thrombosis with myocardial ischemia or free rupture and fatal tamponade [8]. These facts have to be taken seriously when managing such a rare case. The management remains a debatable topic between cardiologists and surgeons as no formal treatment guidelines exist. Boyer et al. proposed a management strategy for those patients presenting with an acute coronary syndrome with regular size CAAs [5]. This is based on the available literature and the guidelines of the American Cardiology College/American Heart Association. According to his study, surgical revascularization is recommended when (a) a CAA involves the main stem of the left coronary artery, (b) multivessel coronary artery disease is identified, (c) a giant CAA is present—diameter increase by 4 times the adjacent normal vessel segment, and (d) when other cardiac conditions mandate surgery. According to these suggestions, our patient met three out of the four criteria, so surgery was justified. We decided to dissect open and explore the aneurismal sacs. This exploration permitted the evaluation of the lumen for the distal end of the coronary vessel. The point for the peripheral anastomosis was decided at this time. Furthermore, thrombus material in the coronary lumen which could explain the angina symptoms was removed. The open ends were ligated both proximally and distally as Mariscalco et al. suggested [9]. The multicoronary involvement mandates multigraft bypass using both arterial and venous grafts and standard techniques. Both antegrade and retrograde cardioplegia was utilised, however, solely retrograde could be an option. The immediate success of the operation is reflected on the fact that minimal inotropic support was required postoperatively and the patient remained symptom-free for the following two years of follow-up. It is unknown why such large findings were missed on echocardiography at the acute admission. As atherosclerosis is reported as the commonest cause of CAAs, it is possible that in patients presenting with cardiac complaints this unusual pathology may not be suspected. This may either go unnoticed in initial assessment—as in our case—or present a differential diagnostic dilemma, e.g., cardiac or mediastinal masses misleading the decision for the clearly beneficial surgical repair. Thus, case reports as the one presented may be of informative value for the clinical doctor and surgeon if ever comes across with such a rare and surprising finding highlighting the need for a high suspicion index and careful clinical interpretation.
